# Comparative Analysis of Clinical and Genomic Characteristics of Hypervirulent Klebsiella pneumoniae from Hospital and Community Settings: Experience from a Tertiary Healthcare Center in India

**DOI:** 10.1128/spectrum.00376-22

**Published:** 2022-08-31

**Authors:** Stephen Raj, Tanya Sharma, Dibyabhaba Pradhan, Sonu Tyagi, Hitender Gautam, Harpreet Singh, Seema Sood, Benu Dhawan, Bimal Kumar Das, Arti Kapil, Rama Chaudhry, Sarita Mohapatra

**Affiliations:** a Department of Microbiology, All India Institute of Medical Sciences, New Delhi, India; b ICMR AIIMS Computational Genomics Centre, Division of Biomedical Informatics, Indian Council of Medical Research, New Delhi, India; Universidad de Buenos Aires, Facultad de Farmacia y Bioquímica

**Keywords:** hypervirulent *Klebsiella pneumoniae* (hvKp), health care settings, community settings, whole-genome sequencing (WGS), antimicrobial resistance (AMR), virulence genes

## Abstract

Hypervirulent Klebsiella pneumoniae (hvKp) is a hypermucoviscous phenotype of classical Klebsiella pneumoniae (cKp) that causes serious infections in the community. The recent emergence of multidrug-resistant hvKp isolates (producing extended-spectrum beta-lactamases and carbapenemases) along with other virulence factors in health care settings has become a clinical crisis. Here, we aimed to compare the distribution of virulence determinants and antimicrobial resistance (AMR) genes in relation to various sequence types (STs) among the clinical hvKp isolates from both settings, to reinforce our understanding of their epidemiology and pathogenic potential. A total of 120 K. pneumoniae isolates confirmed by matrix-assisted laser desorption ionization–time of flight mass spectrometry were selected. hvKp was phenotypically identified by string test and genotypically confirmed by the presence of the *iucA* gene using PCR. Molecular characterization of hvKp isolates was done by whole-genome sequencing (WGS). Of the K. pneumoniae isolates, 11.6% (14/120) isolates were confirmed as hvKp by PCR (9.1% [11/120] string positive and 3.3% [4/120] positive by both methods); these were predominantly isolated from bloodstream infection (50%, 7/14), urinary tract infection (29%, 4/14), and respiratory tract infection (21%, 3/14). For all 14 hvKp infections, for 14.2% the source was in the community and for 85.7% the source was a health care setting. Two virulent plasmids were identified by WGS among the hvKp isolates from both settings. K64 was found to be the commonest capsular serotype (28.5%, 4/14), and ST2096 was the most common ST (28.5%, 4/14) by WGS. Two new STs were revealed: ST231 (reported to cause outbreaks) and ST43. The genome of one isolate was determined to be carrying AMR genes (*bla*_CTX-M-15_, *bla*_NDM-1_, *bla*_NDM-5_, *bla*_OXA-181_, *bla*_OXA-232_, etc.) in addition to virulence genes, highlighting the clonal spread of hvKp in both community and health care settings.

**IMPORTANCE** To date, studies comparing the genomic characteristics of hospital- and community-acquired hvKp were very few in India. In this study, we analyzed the clinical and genomic characteristics of hvKp isolates from hospital and community settings. ST2096 was found as the most common ST along with novel STs ST231 and ST43. Our study also revealed the genome is simultaneously carrying AMR as well as virulence genes in isolates from both settings, highlighting the emergence of MDR hvKp STs integrated with virulence genes in both community and health care settings. Thus, hvKp may present a serious global threat, and essential steps are needed to prevent its further dissemination.

## INTRODUCTION

Klebsiella pneumoniae is a Gram-negative, capsulated, facultative anaerobe belonging to the family *Enterobacterales*. It is one of the clinically significant Gram-negative bacilli that causes a wide range of nosocomial and community-acquired infections. Recently, it gained more attention due to its higher propensity for the acquisition of antimicrobial resistance (AMR) genes, especially those for carbapenemases (1, 2). Hypervirulent K. pneumoniae (hvKp) is another hypermucoviscous pathophenotype of classical K. pneumoniae (cKp) that emerged recently owing to its high morbidity and mortality (1, 2). It originated from community settings, causing serious infections in the immunocompetent population due to the acquisition of virulence genes during its evolution (1). Recent reports have shown the emergence of hospital-acquired hvKp infections in different clinical settings (3, 4). The spectrum of infections may vary from self-limiting to disseminated life-threatening invasive infections (5). The common infections reported in the community setting are pyogenic liver abscess with metastatic complications, bacteremia, and pneumonia, and the common health care-associated infections are ventilator-associated pneumonia, central line-associated bloodstream infections, catheter-associated urinary tract infection, and surgical site infections (SSIs) (5). Apart from its infective role, hvKp has also been observed to persist asymptomatically as a colonizer and to be disseminated in the community (6). Initial cases of hvKp infections were documented in the Asian Pacific Rim, and later cases were reported from all over the world, including Australia, America, Europe, and Africa (1). A large virulence plasmid of K. pneumoniae (pLVPK-like) encoding several virulence genes, including genes for capsular polysaccharide synthesis regulators (*rmpA* and *rmpA2*), iron acquisition systems (*iucA* and *iutA*), and siderophores, has been detected in most hvKp isolates (1). Among these genes, siderophores, enterobactin, and yersiniabactin are produced by all K. pneumoniae strains, whereas aerobactin and salmochelin are mostly harbored by hvKp strains (7). Invasive hvKp isolates are predominantly found to harbor K1 and K2 capsular types with clonal group 23 (CG23) and ST23, ST26, ST57, and ST1633 (8).

A string test can be used for the phenotypic identification of hvKp isolates, with sensitivity ranges from 51% to 98% (9). Molecular identification of hvKp strains is done by the detection of virulence genes, i.e., *iucA*, *rmpA*, *rmpA2*, and *magA* (9). *iucA*, encoding aerobactin, is considered one of the most accurate virulence markers for the identification of hvKp isolates (accuracy of 0.96, sensitivity of 0.99, specificity of 0.94) (9).

Currently, convergent hvKp strains carrying both virulence and AMR genes are being reported from health care settings, and these present an alarming threat to clinicians. In contrast to the earlier susceptible strains, these hvKp isolates are more drug resistant, possibly due to the horizontal transfer of genes among hvKp and cKp isolates (10). Epidemiological research on recent clinical isolates from Southeast Asian countries indicated a high probability of spread of resistant hvKp strains globally in the near future (11). Hence, early recognition of this hypervirulent strain, including its resistance determinants, is a priority concern globally. There have been very few reports in the literature comparing the molecular characteristics of hvKp isolates from hospitals and community settings (12). To address this knowledge gap, the present study aimed to find out the current occurrence of hvKp in a tertiary care health center in India. We also analyzed the clinical outcomes with the genomic characterizations and delineated different serotypes, virulence-associated markers, and antimicrobial drug resistance genes among the hvKp isolates from both hospital and community settings by using whole-genome sequencing (WGS).

## RESULTS

### Demographic profile and clinical characteristics.

A total of 120 patients with culture-positive results for K. pneumoniae from different clinical specimens over a period of 2 years were included in the present study. The demographic and clinical characteristic details could only be collected for 108 patients of the total 120. Fourteen of 120 the K. pneumoniae isolates (11.6%) were confirmed as hvKp. Among these, two isolates (S59 and S61) were from the community and 12 (the rest of the 14 isolates) were from the hospital setting. Demographic details of patients are shown in Table 1. In the current study, the cKp- and hvKp-positive cases were predominantly males. Compared to cKp, hvKp patients predominantly belonged to age groups 19 to 30 years and 31 to 55 years. Prevalence of hvKp in extreme age groups was observed less frequently in our study. When we compared both groups, cKp infection was more often associated with blood malignancy, diabetes mellitus, and chronic kidney disease (CKD) than other underlying conditions, whereas patients with hvKp infection were observed to have more solid organ malignancy (35.7% versus 11.3%), and the association was statistically significant (*P* = 0.014). None of the patients infected with hvKp from either the hospital or community was found to have any preexisting hepatobiliary disease in the current study.

**TABLE 1:** Demographic data of patients infected with hvKp and cKp

Demographic group	% (no.) of patients infected with:			
	hvKp (*n* = 14)		cKp (*n* = 94)	
	CA (*n* = 2)	HA (*n* = 12)	CA (*n* = 28)	HA (*n* = 66)
Age (yrs)				
0–5	0	8% (1)	10.7% (3)	19.6% (13)
6–18	0	8% (1)	21.4% (6)	4.5% (3)
19–30	50% (1)	33.3% (4)	14.2% (4)	12% (8)
31–55	50% (1)	33.3% (4)	35.75% (10)	34.8% (23)
>55	0	16.6% (2)	17.8% (5)	28.7% (19)
Sex				
M:F	1:1	3:1	1:1	1.2:1
Male	50% (1)	75% (9)	50% (14)	56% (37)
Female	50% (1)	25% (3)	50% (14)	44% (29)
Sample				
Urine	0	33.3% (4)	53.5% (15)	30.3% (20)
Blood	0	58.3% (7)	25% (7)	28.7% (19)
Respiratory	100% (2)	8.3% (1)	3.5% (1)	24% (16)
Pus and tissue	0	0	17.8% (5)	16.6% (11)
Underlying condition				
Diabetes	0	16.6% (2)	35.7% (10)	19.6% (13)
CKD	0	8% (1)	10.7% (3)	12% (8)
Solid organ malignancy^*a*^	50% (1)*	33.3% (4)*	7% (2)	15% (10)
Blood malignancy	0	8%(1)	7% (2)	10.6% (7)
Hepatobiliary disease	0	0	17.8 (5)	9% (6)
Outcome				
Death	0	33.3% (4)	17.8% (5)	37% (25)
Discharge	100% (2)	66.6% (8)	82% (23)	62% (41)

a Asterisks indicate a significant association between solid organ malignancy and hvKp infection (*P* < 0.05).

Approximately 7 of 14 hvKp isolates were recovered from blood, 4 were recovered from urine, and 3 were from respiratory specimens. In contrast, the majority of cKp isolates were isolated from urine (37%), followed by blood (31%), pus (16%), and respiratory specimens (16%). Seven patients with hvKp infection had bacteremia, and four were receiving immunosuppression due to underlying conditions of malignancy (Table 2). Ten patients had external devices, e.g., a central line catheter, urinary catheter, and/or mechanical ventilator. Four patients (28%) expired during their hospital stay (3 of the 7 patients with bacteremia and 1 of the 3 patients with respiratory infection). The death rate was approximately the same in both the hvKp and cKp groups (4/14 [28.5%] versus 30/106 [28%], respectively), with a higher incidence (50%) among the adult patients with hvKp infection. All four patients who expired due to hvKp infection had acquired the infection in the health care facility and were observed to have CKD, diabetes, or blood malignancy as an underlying condition (Table 2).

**TABLE 2:** Clinical characteristics of patients infected with hvKp

Sample no.	String test	*iucA*	Sample	Device(s)^*a*^	Diabetes	Immunosuppression	Source^*a*^	Outcome
1	Neg	+	Blood	CL		Yes	HA	Expired
2	Neg	+	Urine	UC			HA	Improved
3	Pos	+	Urine	VL, UC			HA	Improved
4	Pos	+	Blood	VL, CL			HA	Improved
5	Neg	+	Urine	UC			HA	Improved
6	Pos	+	Respiratory			Yes	CA	Improved
7	Neg	+	Respiratory			Yes	CA	Improved
8	Neg	+	Urine	VL	Yes		HA	Improved
9	Neg	+	Blood	UC, CL			HA	Expired
10	Pos	+	Blood				HA	Expired
11	Neg	+	Blood	CL			HA	Improved
12	Neg	+	Respiratory	VL	Yes	Yes	HA	Expired
13	Neg	+	Blood	CL			HA	Improved
14	Neg	+	Blood			Yes	HA	Improved

a CL, central line; VL, ventilator; UC, urinary catheter; HA, healthcare associated; CA, community acquired.

### Identification of hvKp by string test and PCR for the *iucA* gene.

Of the total 120 K. pneumoniae isolates, 11 were positive for the string test and 14 for *iucA* using PCR. Four of 14 (28.5%) hvKp isolates were string test positive, with string lengths ranging from 5 to 10 mm, whereas 7 of 106 (6.6%) cKp isolates were positive for the string test, with a string length of more than 15 mm in one of them. Considering PCR as the gold standard, the sensitivity and specificity of the string test in the present study were 28.7% and 93.4%, respectively. The association of the hypermucoviscous phenotype with hvKp was statistically significant (*P* = 0.024).

### Antimicrobial resistance among hvKp and cKp isolates.

The AMR rate was observed more frequently among the hvKp isolates than among cKp isolates, with resistance rates in hvKp versus cKp isolates, respectively, observed against third-generation cephalosporins (92% versus 83%), fluoroquinolones (92% versus 75%), carbapenems (78% versus 63%), and colistin (12.5% versus 2.7%). Nitrofurantoin was tested only for urinary isolates. Seventy-five percent (3/4) of the hvKp isolates from urine were resistant to nitrofurantoin, in comparison to 59% of the cKp isolates.

cKp isolates from the community (community-acquired cKp [CA-cKp]) were more resistant to fluoroquinolones and third-generation cephalosporins than were hvKp isolates from the community (CA-hvKp); however, the total number of CA-hvKp isolates was much smaller in the present study. The rate of resistance was in a similar range for both the hospital-acquired hvKp (HA-hvKp) and HA-cKp isolates, except for colistin, for which higher resistance was observed among the HA-hvKp isolates (Table 3).

**TABLE 3:** Comparative analysis of antimicrobial resistance patterns of hvKp and cKp isolates causing community-acquired or health care-associated infections

Antimicrobial agent(s)	No. (%) of infections			
	Community-acquired infection (*n* = 30)		Healthcare-associated infections (*n* = 78)	
	hvKp (*n* = 2)	cKp (*n* = 28)	hvKp (*n* = 12)	cKp (*n* = 66)
Aminoglycosides	1 (50%)	13 (46.4%)	11 (91.6%)	51 (77%)
Beta-lactam and beta-lactamase inhibitors	1 (50%)	11 (39%)	11 (91.6%)	51 (77%)
3rd-generation cephalosporins	1 (50%)	21 (75%)	12 (100%)	58 (87%)
Fluoroquinolones	1 (50%)	18 (64%)	12 (100%)	54 (81%)
Nitrofurantoin	0	6/13 (46%)	3/4 (75%)	16/19 (84%)
Carbapenem	0	10 (35.7%)	9 (75%)	47 (71%)
Colistin	0	0	1 (8%)	1 (1.5%)

### Result of WGS analysis.

**(i) Qualitative assessment results for the assembled genomes.** The whole-genome assemblies were evaluated based on the *N*_50_, *L*_50_, number of contigs, and genome completeness. All the isolates except one (S62) showed >96% genome completion. Three references selected were used to evaluate the quality of the draft genomes using QUAST. The assemblies with the highest genome fraction, high *N*_50_, and fewer contigs are reported. The mean *N*_50_ value for all the isolates was ≈246,926. The genome size of the references was ≈5.4 to 5.5 Mb. The size of the genomes ranged from 5.3 Mb to 7.4 Mb, with the largest contig length ranging from 71 kb to 1 Mb.

**FIG 1:**
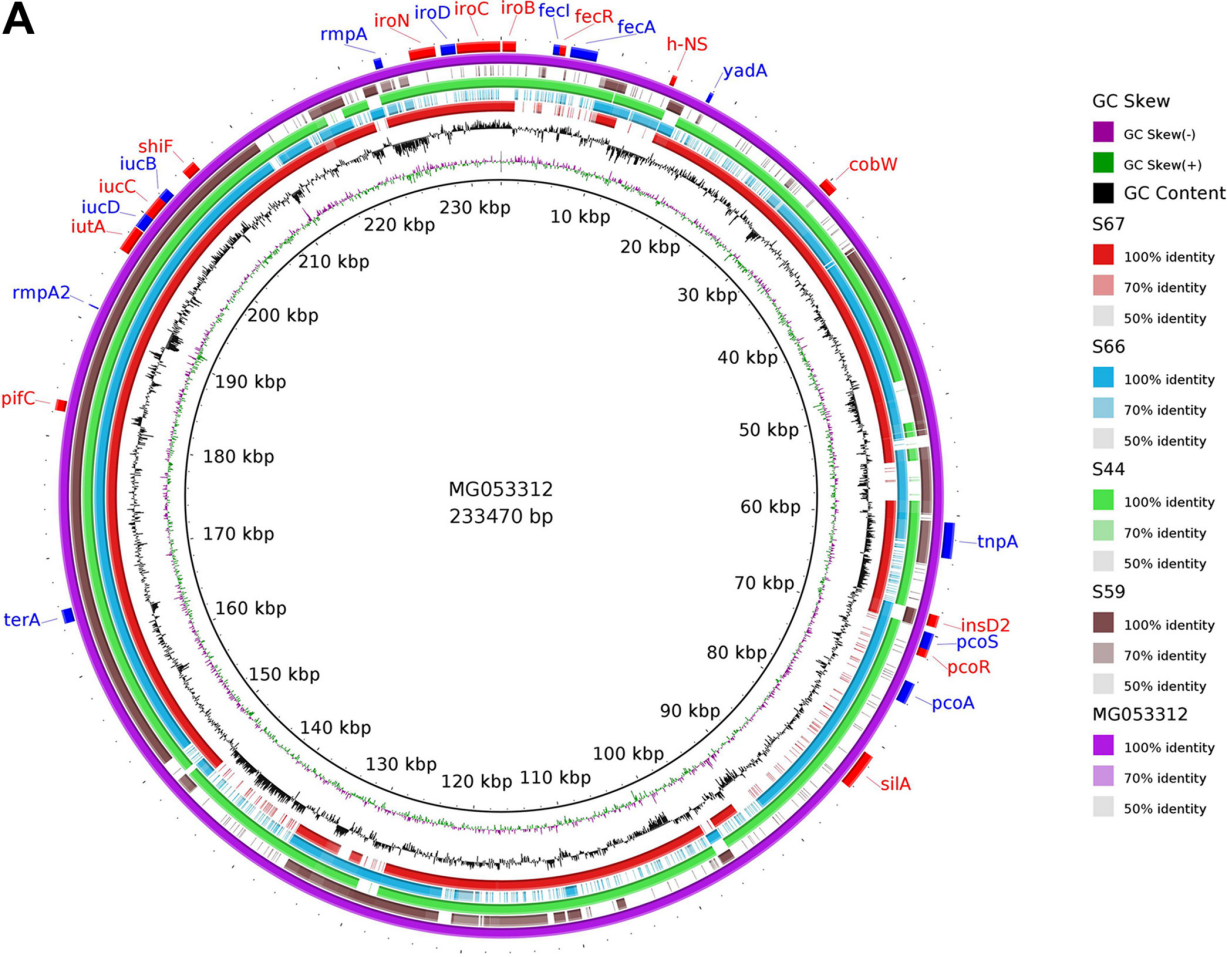

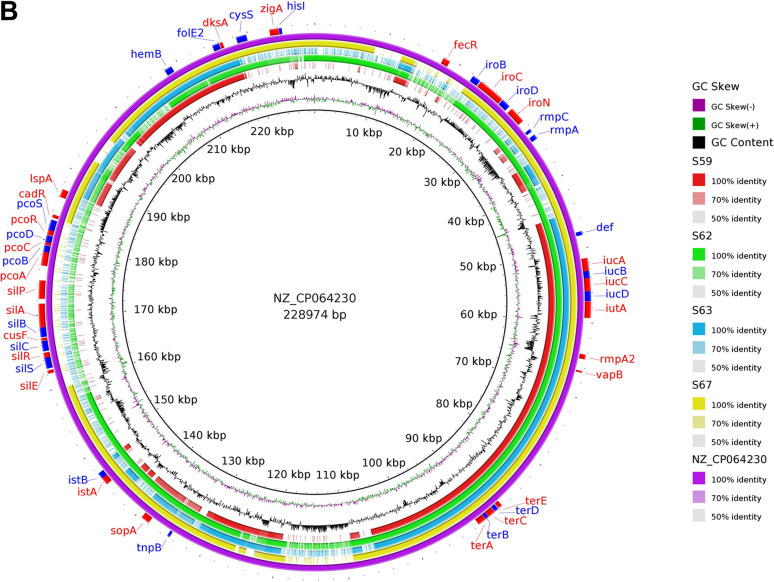
Sequence alignment of hvKp reference plasmids and the putative assembled plasmids. (A) The purple ring represents the reference hvKp plasmid pVir-CR-HvKP267 (accession number MG053312). The blastn comparison was done between the putative assembled plasmids and the complete sequence of the reference plasmid. The red, blue, green, and brown rings represent the samples S67, S66, S44, and S59, respectively. The outer ring with the red and blue arcs signifies the annotation of pVir-CR-HvKP267 and represents the genes present in the plasmid sequences. (B) Comparison between the hvKp plasmid pJX6-1 and the assembled plasmids, done using blastn with pJX6-1 as the reference. The yellow, blue, green, and red rings denote the samples S67, S63, S62, and S59, respectively. The red and blue arcs represent the genes in the reference plasmid.

**(ii) Plasmid identification.** Two hypervirulent plasmids, namely, pVir-CR-HvKP267 (NCBI accession number MG053312) and pJX6-1 (NCBI accession number NZ_CP064230) were identified in isolates S67, S66, S44, and S59 and in S67, S63, S62, and S59, respectively (Fig. 1). The full lengths of the plasmids pVir-CR-HvKP267 and pJX6-1 were ≈233 kbp and ≈228 kbp, respectively.

Plasmid sequences of pVir-CR-HvKP267 contained *iucB*, *iucC*, *iucD*, *iutA*, *rmpA2*, and *terA* genes in all of the isolates, whereas *iroN*, *iroD*, *iroC*, and *iroB* were found in S44 and S67 isolates only. No AMR genes were detected in the plasmids. The pVir-CR-HvKP267 plasmid sequence was highly similar to that of the virulence plasmid pLVKP (large virulence plasmid of K. pneumoniae; accession number AY378100) reported earlier from Singapore in a patient with liver abscess (13). pLVKP has also been reported from China, emphasizing the emergence of drug resistance genes in K. pneumoniae isolates. Moreover, the pLVKP plasmid in our study was present in the hvKp isolates from both hospital and community settings.

**(iii) O-antigen genotyping, capsular genotyping, and sequence types of the isolates.** Both lipopolysaccharide O-antigen and capsular polysaccharide K-antigen types play a crucial role in the virulence of Klebsiella pneumoniae with important clinical and epidemiological significance (14, 15). In the current study, the O1 locus was found in 12 isolates, of which 9 had the O1v1 locus and 2 had the O1v2 locus. The O2 (O2v1) locus was found in one isolate, while the O3 (O3/O3a) locus was found in two more hvKp isolates.

Capsular genotyping of the 14 hvKp isolates predicted the different K types. K64 and K2 were observed in four hvKp isolates and were the most common capsular types. Other less common capsular types were K1, K51, K10, and K30. The sequence types were predicted based on 7 housekeeping genes, namely, *gapA*, *infB*, *mdh*, *pgi*, *phoE*, *rpoB*, and *tonB*. Kleborate and MLST 2.0 indicated the novel allele *phoE* for isolate S67, with 99.8% identity. Most of the hvKp strains belonged to ST2096 (4/14), and one isolate was found to have ST23. The most common ST and K type combination observed in the current study was ST2096-K64 (*n* = 4), and the remaining combinations were ST23-K1, ST15-K2, ST147-K10, ST336-K64, ST2857-K1, ST231-K51, ST86-K2, and ST43-K30.

**(iv) Virulence genes.** WGS revealed the presence of various virulence genes in the isolates, including genes for allantoin metabolism (*allA*, *allB*, *allC*, *allD*, *allE*, *allR*, and *allS*), iron uptake (*kfuA*, *kfuB*, *kfuC*, and *fyuC*), and siderophores such as aerobactin (*iucA*, *iucB*, *iucC*, *iucD*, and *iutA*), salmochelin (*iroB*, *iroC*, *iroD*, *iroE*, and *iroN*), enterobactin (*entA*, *entB*, *entC*, *entD*, *entE*, *entF*, *entH*, and *entS*), and yersiniabactin (*ybtA*, *ybtE*, *ybtP*, *ybtQ*, *ybtS*, *ybtT*, *ybtU*, *ybtX*, and *irp*), along with different AMR genes, such as *bla*_CTX-M-15_, *bla*_NDM-1_, *bla*_NDM-5_, *bla*_OXA-181_, and *bla*_OXA-232_.

All hvKp isolates (100%) in this study were found to carry the *iutA* gene, and 92.8% (13/14) carried the *iucA*, *iucB*, and *iucC* genes. The gene encoding salmochelin was detected in 5 hvKp isolates. Approximately 78.5% (11/14) of the total hvKp isolates carried the *rpmA* gene, which is responsible for the hypermucoviscous phenotype, and 93% (13/14) carried *rmpA2*. In addition, genes encoding enterobactin, such as *entA*, *entB*, *entC*, *entD*, *entF*, *entH*, and *entS*, were detected in all isolates. The *entB* gene was present as a variant *entB1* and *entB2* in one isolate, *entC* as *entC1* and *entC2* in one isolate, and *entD* as *entD1* and *entD2* in two isolates. Genes for iron uptake, such as *kfuA* and *kfuB*, were detected in 10 (71.4%) isolates. Genes encoding fimbriae, such as the *mrkA*, *mrkB*, *mrkC*, and *mrkD* genes, were detected in 10 (71.4%) hvKp isolates.

### Variant analysis.

**FIG 2:**
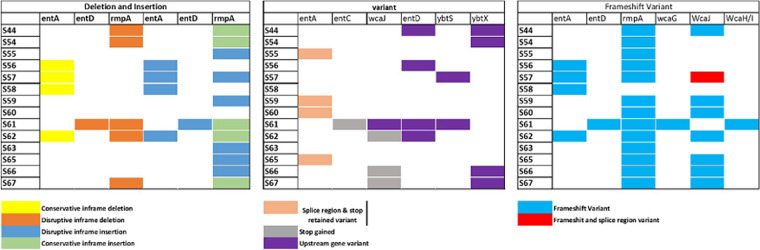
Types of variants present in the hvKp isolates. The colored block represents the presence of the genetic variants in the corresponding isolates.

Classically, gene variants are of six types: deletion, insertion, splice region and stop retained, stop gained, upstream gene variant, and frameshift variant. The total number of variants with a high putative effect of deleteriousness found in isolates were frameshifts (4,995), start-lost (132), and stop-gained (478). The variants with moderate deleteriousness were a conservative in-frame deletion (227), conservative in-frame insertion (302), disruptive in-frame deletion (378), and disruptive in-frame insertion (328). The top 10 highly mutated genes were *KP1_0403*, *KP1_4102*, *fimD*, *mobB*, *wzi*, *wzc*, *KP1_0410*, *ardC*, *wza*, and *KP1_4970.* In the present study, most of these types of variations were found in *ent* genes (*entA*, *entD*, and *entC*) (Fig. 2). The *rmpA* gene was found mutated in most of the isolates carrying a conservative in-frame insertion or deletion (except for S56, S58, and S60) and a frameshift variation in all the isolates. Frameshift mutation was also discovered in *wcaJ* in all isolates except S58. See Table S3 in the supplemental material for a summary of functions of the genes listed above.

**FIG 3:**
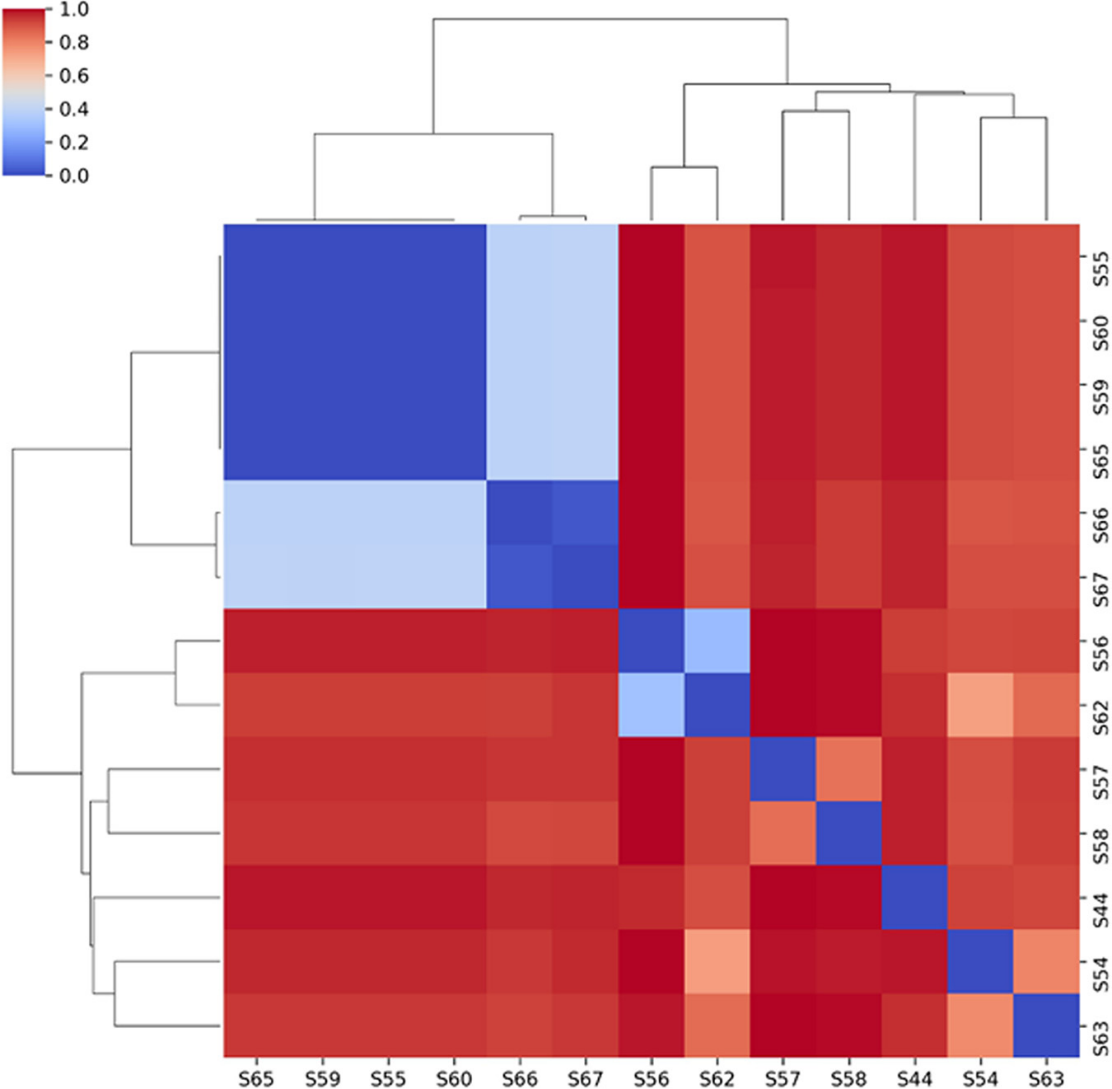
ClusterMap showing pairwise SNP distances and divergence between all the hvKp isolates except for one (S61). The color range from dark blue to dark red indicates the least SNP divergence to higher SNP divergence, respectively. The scale bar represents the percentage of SNP divergence with the color range.

The ClusterMap showing evolutionary distance based on mutations was built using CSI phylogeny (Fig. 3). According to the ClusterMap, S56 was at a greater evolutionary distance from most of the isolates (S63, S54, S58, S57, S67, S66, S65, S59, S60, and S65). Similarly, S57 and S58 had higher single-nucleotide polymorphism (SNP) divergence with S63, S54, S62, and S56.

### Antimicrobial resistance gene identification and analysis.

**FIG 4:**
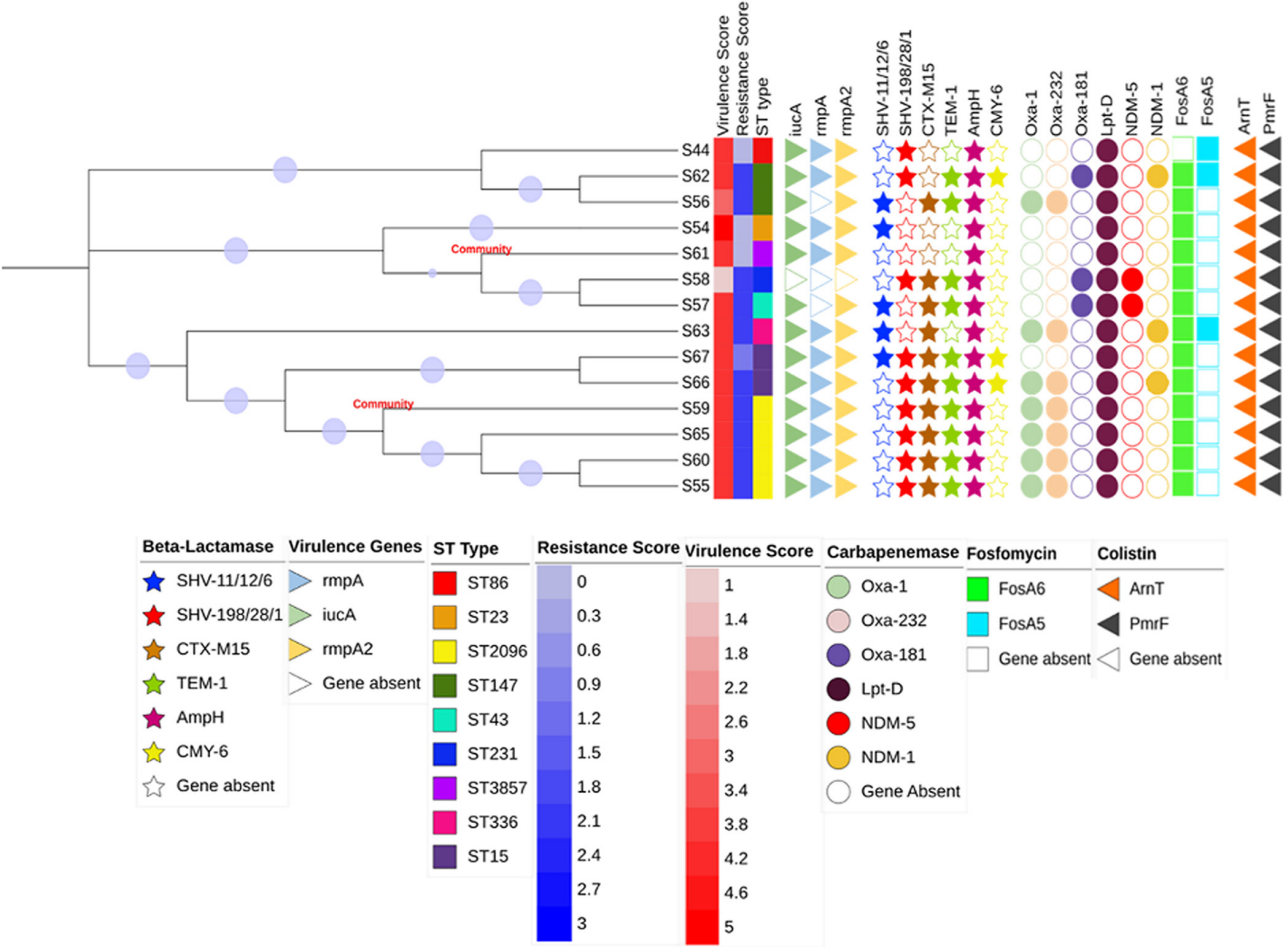
Phylogenetic tree based on MLST and core genome alignment. The community-acquired hvKp isolates are S59 and S61 whereas rest are hospital-acquired hvKp isolates. The bootstraps are shown as blue circles on the branches.

The distribution of different AMR genes, including those for extended-spectrum beta-lactamases (ESBLs) and carbapenemases, among the hvKp isolates is shown in Fig. 4. All of the hvKp isolates carried at least one AMR gene, with 85.7% of isolates being multidrug resistant (MDR). The *bla*_CTX-M-15_ gene (ESBL type) was the most common beta-lactamase gene (*n* = 11/14, 78.5%), followed by *bla*_SHV_ (ESBL type; *n* = 10/14, 71.4%). Three patients of the 11 harboring *bla*_CTX-M-15_ expired during the course of the disease. The variants detected for *bla*_SHV_ were *bla*_SHV-1_, *bla*_SHV-6_, *bla*_SHV-11_, *bla*_SHV-12_, *bla*_SHV-22_, *bla*_SHV-28_, *bla*_SHV-105_, and *bla*_SHV-198_. Among the carbapenemases, *bla*_OXA_ was the dominant genotype (*n* = 10/14, 71.4%), followed by *bla*_NDM_ (*n* = 5/14, 35.7%). Five of 14 hvKp isolates were positive for *bla*_NDM_, of which three were *bla*_NDM-5_ and two were *bla*_NDM-1_. The variants found for *bla*_OXA_ were *bla*_OXA-1_, *bla*_OXA-181_, and *bla*_OXA-232_. Thus, 35.7% (5/14) of hvKp isolates cocarried *bla*_CTX-M-15_, *bla*_SHV_, *bla*_OXA_, and *bla*_NDM_ and were found as the predominant MDR hvKp genotype. One of the CA-hvKp isolates carried *bla*_SHV_, *bla*_CTX-M-15_, *bla*_TEM-1_, *bla*_OXA-1_, and *bla*_OXA-232_ in combination. All the hvKp isolates carried fosfomycin resistance genes (for FosA6 or FosA5). Resistance to aminoglycosides was also detected by the presence of the genes *aac(6′)-lb* and *aadA2*. At least two virulence genes were detected in all the resistant hvKp isolates, whereas the susceptible CA-hvKp isolates carried the majority of virulence genes, including *iucA*, *rmpA*, and *rmpA2*.

### Phylogenetic analysis and comparison of virulence and AMR genes.

Based on the multilocus sequence typing (MLST) of 14 hvKp isolates and alignment of the core genome, a phylogenetic tree was constructed (Fig. 4). The isolate-ST combinations S54-ST23, S61-ST3857, S58-ST231, and S57-ST43 formed a single clade. Another clade was formed by isolates having ST15 (S67 and S66), ST2096 (S59, S65, S60, and S55), and ST336 (S63). Isolate S59, which came from a community setting, was seen in a close phylogenetic relationship with S65, S60, and S55. Another community isolate, S61, formed a subclade with isolates S58 and S57 depicting close evolutionary ties. ST2096, the most common ST (28.5%), was detected in both HA-hvKp and CA-hvKp isolates (S59, S65, S60, and S55).

## DISCUSSION

hvKp is an emerging pathophenotype reported in the community setting and has recently caused infections in health care settings, with higher virulence and mortality rates (3, 16). In this study, we analyzed the clinical and molecular characteristics of hvKp isolates from both community and hospital settings to get an idea about their commonality and the emergence of new threats.

In our study, 14 hvKp were identified out of 120 (11.6%) K. pneumoniae isolates, of which 14.2% (2/14) were from the community and 85.3% (12/14) were from the hospital. Approximately half (7/14) of the hvKp-infected patients had bloodstream infections, four had urinary tract infections, and three had respiratory tract infections. Earlier studies reported that most of the hvKp isolates from patients with liver abscesses caused high mortality (1). In contrast, none of the hvKp isolates in the current study was associated with hepatobiliary disease, and both patients with community-acquired respiratory infection survived. Moreover, the clonal relatedness of the strains between the hospital and community settings depicted possible ongoing transmission, which would lead to high heterogeneity (12).

Recent studies have shown an increased incidence of hvKp infections in health care settings, with incidence rates ranging from 37.8% to 46.6% (3, 16). Convergence of both virulence and resistance genes among the hvKp isolates will pose a threat to the population. One study found that 46% of the total SSIs were caused by hvKp, of which 15% were ESBL producers and 7% were resistant to carbapenem (4). Moreover, the emergence of MDR hvKp isolates has been reported, including resistance to colistin, which is used as one of the last-line antibiotics during Gram-negative bacterial infection management (17). In contrast, a few studies have shown less uptake of resistance genes by hvKp isolates due to the presence of an increased number of capsular genes (18). A study by Shankar et al. from India compared health care-associated and community-acquired hvKp isolates and showed more susceptibility against all beta-lactam antibiotics and absence of most of the AMR genes (12). In contrast, the current study showed a very high rate of resistance in both HA-hvKp and CA-hvKp isolates, although the total number of isolates in both groups was lower. The rate of MDR hvKp isolates in our study was 85.7%, and 75% of the total HA-hvKp isolates showed resistance against carbapenem. Moreover, the presence of *bla*_CTX-M-15_, *bla*_SHV_, *bla*_OXA_, and *bla*_NDM_ was found simultaneously in 35.7% of total hvKp isolates, including one isolate from the community. Phenotypically, no resistance was observed against carbapenem, nitrofurantoin, or colistin in any of the CA-hvKp isolates in our study. Sanikhani et al. showed a high percentage of MDR hvKp (76.6%), with the presence of *bla*_CTX-M-15_ (76.5%), *bla*_SHV_ (80.4%), *bla*_OXA-48_ (53.9%), and *bla*_NDM-1_ (32.3%) (18). Our study results are consistent with those findings. Moreover, the presence of *bla*_CTX-M-15_, *bla*_TEM-1_, *bla*_OXA-1_, and *bla*_OXA-232_ in the CA-hvKp isolates highlights the emerging resistance in the community settings along with the virulence. However, more studies with higher numbers of hvKp isolates from both settings in different geographical areas are required to estimate the actual burden of this problem.

It was observed that there was the existence of both resistance genes and virulence genes in three of the HA-hvKp isolates. *bla*_NDM_ along with other *bla*_OXA-232_ carbapenemase genes were present in the high-risk clones. The high-risk clones of ST15 and ST36 (isolates S67 and S63, respectively) carried *bla*_NDM-1_, *bla*_OXA-232_, and *rmpA2/rmpA*, and ST43 (isolate S57) carried *bla*_NDM-_*_5_* and *rmpA2*. The patients infected with high-risk clones ST15 and ST43 expired. This convergence of AMR genes by the horizontal acquisition of mobile genetic elements and virulence-associated plasmids (pLVPK, pVir-CR-HvKP267, and pJX6-1) leads to the emergence of highly resistant hvKp with high pathogenicity. This poses a serious public health threat in both communities and hospitals.

Two hypervirulent plasmids, namely, pVir-CR-HvKP267 (NCBI accession number MG053312) and pJX6-1 (NCBI accession number NZ_CP064230), were identified in both CA-hvKp and HA-hvKp isolates. These plasmids were found to carry most of the virulence genes encoding aerobactin (*iucABCD* and *iutA*) salmochelin (*iroBCDN*), and regulator of mucoid phenotype (*rmpA* and *rmpA2*). Siderophore systems are important for bacterial pathogenicity, as they help bacteria to scavenge iron from host transport proteins, allowing them to survive and proliferate in the host (19). Aerobactin plays an important role in both *in vivo* and *ex vivo* survival of hvKp compared to other siderophores (1). Aerobactin has been identified as the most prevalent siderophore in hvKp (1, 9). Our study showed the presence of *iutA* universally among all the isolates, whereas 92.8% of isolates harbored *iucA*, *iucB*, and *iucC*. The copresence of *iutA*, *iucA*, *rmpA*, and *rmpA2* was observed in most of the hvKp isolates in our study. Although the *iucA* gene was present in all the isolates by PCR, it was observed to be absent in S58 by WGS analysis. It was also observed that many other virulence genes were absent in this isolate, which could be possibly due to either sequencing error or the presence of partial gene fragments. Nearly 71.4% of the hvKp isolates were found to contain *mrkA* for fimbriae, *mrkB* and *mrkC* for assembly of fimbriae, and *mrkD* for adhesion, and these were mostly observed in patients with invasive medical devices.

ST2096 was the most common ST observed in our study, harboring *iucA*, *rmpA*, and *rmpA2* with MDR genes (*bla*_OXA-232_, *bla*_OXA-1_, *bla*_CTX-M-15_, and *bla*_SHV_) and found in both hospital and community hvKp isolates. The clonal group CG23, which comprises the sequence types ST23, ST26, ST57, and ST163, was mostly responsible for the hypervirulent phenotypes (8). These nosocomial clones have rapidly acquired a virulence plasmid in recent years, indicating that they are now primed for nosocomial and health care-related epidemics. This highlights the convergence of AMR genes and virulence genes among hospital and community hvKp isolates. Previous studies showed ST23 and ST11 as the predominant STs associated with hvKp infection and the evolution of MDR strains (20). However, in the present study, only one isolate was ST23, with the *iucA*, *rmpA*, and *rmpA2* genes without any carbapenem resistance genes. In addition, one of the hvKp isolates in the current study was found to have ST231, which contains *bla*_NDM-5_ and *iutA* and is known to have caused an outbreak in a hospital (21). To the best of our knowledge, this is the first study to report the presence of ST231 and ST43 in hvKp isolates. Therefore, strict monitoring and surveillance of hvKp infection is highly essential to restrict this convergence, by prompt identification and strictly implementing infection control measures.

### Limitations.

This study has certain limitations. First, it is a single-center study. The clinical affection might be different in different geographical regions. However, the majority of clinical characteristics reported in our study are consistent with those of earlier published studies. Second, we only targeted the *iucA* gene for the confirmation of hvKp isolates in this study. Other studies have used virulence marker genes *iutA* and *rmpA2* for detection of hvKp, which might be helpful to identify more hvKp isolates. Third, the number of isolates from community settings was lower in this study, and this might have been due to the ongoing pandemic. Genomic study with more isolates from different geographical areas might be helpful to understand disease pathogenesis and phylogenetic relationships.

### Conclusion.

Here, we have reported the whole-genome sequencing results of 14 hvKp isolates from both community and hospital settings and compared their molecular characteristics. ST2096 was found to be the most common serotype associated with hvKp isolates from both hospital and community settings, along with the new ST ST231 and high-risk clones ST15, ST36, and ST43. Our study highlights the emergence of drug-resistant hvKp sequence types carrying various virulence genes in both the community as well as hospital setting, which may lead to fatal outcomes in both settings. Therefore, there is an urgent need to identify hvKp infections, establish the biomarkers for its accurate and prompt identification, and implement strict infection prevention protocols to restrict its dissemination.

## MATERIALS AND METHODS

### Study design, ethics, and consent.

This was a prospective, cross-sectional study conducted in the Department of Microbiology, from August 2019 to June 2021. Ethical permission for the study was obtained from the institutional ethical committee (approval number IECPG-441/27.06.2019). Informed consent was obtained from all the patients who participated in this study. Biological specimens, i.e., blood, urine, respiratory specimens (bronchoalveolar lavage fluid, endotracheal aspirate, and sputum), pus, and tissue biopsy specimens of the patients attended to at the hospital with a clinical diagnosis were included in this study and processed as per the standard operative protocol (SOP) of the bacteriology laboratory. All patients with a definite clinical diagnosis where K. pneumoniae was isolated from the clinical specimens using the SOP were included in the study, and patients in which K. pneumoniae isolates were identified as colonizers were excluded from the study.

### Microbiological identification and antimicrobial susceptibility testing of K. pneumoniae isolates.

An infection of the patient that occurred after 48 h of hospital admission was considered hospital acquired, and an infection identified within 48 h of admission was considered community acquired. All the clinical isolates recovered after the bacteriological processing were identified using matrix-assisted laser desorption ionization–time of flight mass spectrometry (bioMérieux, Germany), and antimicrobial susceptibility testing (AST) was performed by Kirby-Bauer disc diffusion test. The antibiotic discs tested were amikacin, ceftazidime, ciprofloxacin, cefotaxime, netilmicin, nitrofurantoin, piperacillin-tazobactam, imipenem, and meropenem. Susceptibility testing for colistin was performed using the broth microdilution method. The AST results were interpreted as per CLSI 2019 guidelines. All the K. pneumoniae isolates were tested in the string test and archived in glycerol stock vials at −80°C for molecular characterization. The patients were followed up for their demographic profile (age, sex), underlying conditions (diabetes mellitus, solid organ malignancy, blood malignancy immunosuppression, or CKD), presence of any external devices (ventilator, central line, or urinary catheter), and outcome (death and discharge) during the hospital stay. All the archived isolates were checked for the presence of the *iucA* (aerobactin) gene using PCR. The string test was considered positive if the string length from the bacterial colony could be stretched to more than 5 mm using a bacteriological inoculation loop (22). hvKp isolates were phenotypically identified by a positive string test and genotypically confirmed by PCR targeting the *iucA* gene using published primers (47).

### DNA extraction and PCR for *iucA*.

The glycerol stock vials containing K. pneumoniae isolates were taken from −80°C storage and kept at room temperature for 30 min. Subculture was done on 5% sheep blood agar and incubation at 37°C for 18 to 24 h. DNA was extracted from the cultures using the QIAamp DNA Mini extraction kit. PCR for *iucA* was performed for all K. pneumoniae isolates. Primers used for *iucA* PCR were the following: forward, 5-ATAAGGCAGGCAATCCAG-3′; reverse, 5-TAACGGCGATAAACCTCG-3 (Pengwen et al., unpublished). The total volume (25 μL) of the reaction mixture was prepared by adding 2.5 μL of buffer, 0.5 μL of deoxynucleoside triphosphate, 1-μL volumes of forward and reverse primers, 0.3 μL of *Taq* polymerase, 3 μL of extracted DNA, and 16.7 μL of DNase-free water. The PCR conditions were as follows: initial denaturation at 94°C for 10 min, followed by 94°C for 1 min, annealing at 62°C for 1 min, and extension at 72°C for 1 min for a total of 35 cycles. PCR conditions were standardized in the laboratory by pooling all the string-positive K. pneumoniae isolates. The PCR products were visualized using 1% agarose gel electrophoresis along with a ladder, positive control (2,927 bp), and negative control.

### DNA preparation, whole-genome sequencing, and annotation.

Fourteen *iucA*-positive K. pneumoniae isolates were confirmed as hvKp and subjected to WGS. DNA was extracted using a Qiagen kit as per the manufacturer’s instructions. Extracted DNA was quantified using a Nanoquant Infinite M200 Pro by Tecan, Austria. The WGS was performed with the Illumina platform on the S4 flow cell of a NovaSeq 6000 using 150-bp paired-end chemistry.

### NGS read quality control.

The raw sequencing data of 14 hvKp isolates were evaluated using FastQC v0.11.5 (23), MultiQC (24), and Trimmomatic v0.39 (25). Adaptors and low-quality reads were discarded. The trimmed reads were processed for detecting host and microbial contamination using Kraken2 (26). Subsequently, reads identified to be contaminants were excluded.

### Genome assembly.

High-quality reads were used for draft genome assembly using SPAdes v3.15.2 assembler (27). The scaffolds generated for each sample were optimized in SSPACE v2.0 (28). Subsequently, potential misassemblies, gaps, small indels, and single-base differences were corrected with Pilon v1.24 (29). Quality evaluations for the assembled draft genome were performed based on *N*_50_ and *L*_50_ as well as genome fraction coverage using BUSCO v5.1.2 (30) and QUAST v5.0.2 (31). Genome fraction coverage was calculated based on three reference genomes of K. pneumoniae available from the National Center for Biotechnology Information (accession numbers CP012043, FO834906, and AP006725). The scaffolds were ordered using ABACAS v1.03 (32) and annotated in Prokka v1.14.6 (33).

### WGS analysis methods.

The assembled contigs were used for the prediction of AMR genes, plasmids, and sequence types. The prediction of AMR genes was performed using two commonly used tools, CARD v5.2.1 (34) and ResFinder 4.0 (35). Only high-confidence AMR genes observed in CARD (only strict hits) and ResFinder (identity of ≥90%) were included in our study. Furthermore, the virulence genes were also screened with the Big Pasteur database (https://bigsdb.pasteur.fr/klebsiella/) with default settings. STs of the 14 draft genomes were determined using MLST 2.0 (36) of the Center for Genetic Epidemiology. The Kleborate tool (37) was used to determine the K- and O-antigen serotypes in the hvKp isolates. The clustering of 14 hvKp draft genomes based on MLST was also evaluated using core genome-based alignment with the Parsnp tool (38). Phylogenetic relatedness of the 14 isolates was also studied using CSI Phylogeny (39).

All the hvKp isolates were further analyzed for the presence of plasmid sequences by using Plasmid SPAdes (40). A homology search for the assembled plasmid sequences was carried out against the hypervirulent plasmids of K. pneumoniae. The circular ring diagram displaying hvKp plasmid comparisons was plotted using the BLAST Ring Image Generator (41), which uses BLAST to perform genome comparisons and CGView to generate circular genome images (Fig. 1). Seaborn is the powerful python library used for data visualization. The divergence matrix from CSIPhylogeny was used to generate the cluster map with Seaborn (Fig. 3). The phylogenetic tree based on core genome alignment was built using Parsnp and Gingr (38). The Interactive Tree of Life (42) was used to generate the annotated dendrogram (Fig. 4).

### Read mapping and variant calling.

One reference genome of K. pneumoniae was retrieved from the NCBI GenBank database (NCBI accession number AP006725). The sequences were indexed, and the quality-passed reads of each sample were mapped to the reference genomes by using the Burrows-Wheeler aligner (43). The alignment quality was evaluated through sequencing depth and genome coverage using deepTools (44). The binary sequence alignment maps were processed to mark duplicate reads, and variant calling was performed using GATK HaplotypeCaller in haploid mode (45). The variants were filtered using GATK hard-filter parameters to retain variants most likely to be true positive and not sequencing artifacts (45). In addition, all variants were annotated using snpsift (46).

### Statistical analysis.

Statistical analysis was done using SPSS version 26. Descriptive analysis of the entire data was prepared. Statistical analysis was performed by using the chi-square test or Fisher’s exact test for categorical variables. Categorical data were represented as frequencies and percentages. A *P* value of <0.05 was considered statistically significant.

### Data availability.

The raw sequencing data reported in this study have been deposited in the Sequence Read Archive (SRA; https://www.ncbi.nlm.nih.gov/sra) under NCBI BioProject accession PRJNA797874. The genome sequenced files have been submitted to the NCBI SRA under accession numbers SRR17640814, SRR17640813, SRR17640812, SRR17640811, SRR17640810, SRR17640809, SRR17640808, SRR17640807, SRR17640806, SRR17640805, SRR17640804, SRR17640803, SRR17640802, and SRR17640801.

## References

[1] Shon AS, Bajwa RPS, Russo TA. 2013. Hypervirulent (hypermucoviscous) *Klebsiella pneumoniae*: a new and dangerous breed. Virulence 4:107–118. doi:10.4161/viru.22718. 23302790 PMC3654609

[2] Catalán-Nájera JC, Garza-Ramos U, Barrios-Camacho H. 2017. Hypervirulence and hypermucoviscosity: two different but complementary *Klebsiella* spp. phenotypes? Virulence 8:1111–1123. doi:10.1080/21505594.2017.1317412. 28402698 PMC5711391

[3] Liu C, Guo J. 2018. Characteristics of ventilator-associated pneumonia due to hypervirulent *Klebsiella pneumoniae* genotype in genetic background for the elderly in two tertiary hospitals in China. Antimicrob Resist Infect Control 7:95. doi:10.1186/s13756-018-0371-8. 30128143 PMC6091109

[4] Zhao Q, Guo L, Wang L, Zhao Q, Shen D. 2020. Prevalence and characteristics of surgical site hypervirulent *Klebsiella pneumoniae* isolates. J Clin Lab Anal 34. doi:10.1002/jcla.23364. PMC7521332 32424981

[5] Russo TA, Marr CM. 2019. Hypervirulent *Klebsiella pneumoniae*. Clin Microbiol Rev 32. doi:10.1128/CMR.00001-19. PMC6589860 31092506

[6] Lin Y-T, Siu LK, Lin J-C, Chen T-L, Tseng C-P, Yeh K-M, Chang F-Y, Fung C-P. 2012. Seroepidemiology of *Klebsiella pneumoniae* colonizing the intestinal tract of healthy Chinese and overseas Chinese adults in Asian countries. BMC Microbiol 12:13. doi:10.1186/1471-2180-12-13. 22260182 PMC3273430

[7] Paczosa MK, Mecsas J. 2016. *Klebsiella pneumoniae*: going on the offense with a strong defense. Microbiol Mol Biol Rev 80:629–661. doi:10.1128/MMBR.00078-15. 27307579 PMC4981674

[8] Shi Q, Lan P, Huang D, Hua X, Jiang Y, Zhou J, Yu Y. 2018. Diversity of virulence level phenotype of hypervirulent *Klebsiella pneumoniae* from different sequence type lineage. BMC Microbiol 18:94. doi:10.1186/s12866-018-1236-2. 30157774 PMC6116568

[9] Russo TA, Olson R, Fang C-T, Stoesser N, Miller M, MacDonald U, Hutson A, Barker JH, La Hoz RM, Johnson JR, Backer M, Bajwa R, Catanzaro AT, Crook D, de Almeda K, Fierer J, Greenberg DE, Klevay M, Patel P, Ratner A, Wang J-T, Zola J. 2018. Identification of biomarkers for differentiation of hypervirulent *Klebsiella pneumoniae* from classical *K. pneumoniae*. J Clin Microbiol 56 doi:10.1128/JCM.00776-18. PMC6113484 29925642

[10] Choby JE, Howard-Anderson J, Weiss DS. 2020. Hypervirulent *Klebsiella pneumoniae*: clinical and molecular perspectives. J Intern Med 287:283–300. doi:10.1111/joim.13007. 31677303 PMC7057273

[11] Effah CY, Sun T, Liu S, Wu Y. 2020. *Klebsiella pneumoniae*: an increasing threat to public health. Ann Clin Microbiol Antimicrob 19:1. doi:10.1186/s12941-019-0343-8. 31918737 PMC7050612

[12] Shankar C, Veeraraghavan B, Nabarro LEB, Ravi R, Ragupathi NKD, Rupali P. 2018. Whole genome analysis of hypervirulent *Klebsiella pneumoniae* isolates from community and hospital acquired bloodstream infection. BMC Microbiol 18:6. doi:10.1186/s12866-017-1148-6. 29433440 PMC5809863

[13] Yao H, Qin S, Chen S, Shen J, Du X-D. 2018. Emergence of carbapenem-resistant hypervirulent *Klebsiella pneumoniae*. Lancet Infect Dis 18:25. doi:10.1016/S1473-3099(17)30628-X. 29102518

[14] Cortés G, Borrell N, de Astorza B, Gómez C, Sauleda J, Albertí S. 2002. Molecular analysis of the contribution of the capsular polysaccharide and the lipopolysaccharide O side chain to the virulence of *Klebsiella pneumoniae* in a murine model of pneumonia. Infect Immun 70:2583–2590. doi:10.1128/IAI.70.5.2583-2590.2002. 11953399 PMC127904

[15] Fang C-T, Shih Y-J, Cheong C-M, Yi W-C. 2016. Rapid and accurate determination of lipopolysaccharide O-antigen types in *Klebsiella pneumoniae* with a novel PCR-based O-genotyping method. J Clin Microbiol 54:666–675. doi:10.1128/JCM.02494-15. 26719438 PMC4767969

[16] Zhang Y, Zhao C, Wang Q, Wang X, Chen H, Li H, Zhang F, Li S, Wang R, Wang H. 2016. High prevalence of hypervirulent *Klebsiella pneumoniae* infection in China: geographic distribution, clinical characteristics, and antimicrobial resistance. Antimicrob Agents Chemother 60:6115–6120. doi:10.1128/AAC.01127-16. 27480857 PMC5038323

[17] Kaza P, Britto XB, Mahindroo J, Baker S, Nguyen TNT, Mavuduru RS, Mohan B, Taneja N. 2021. Hypervirulent extensively-drug resistant (XDR) *Klebsiella pneumoniae* associated with complicated urinary tract infection in northern India. medRxiv. doi:10.1101/2021.05.15.21256863. 37648492

[18] Sanikhani R, Moeinirad M, Solgi H, Hadadi A, Shahcheraghi F, Badmasti F. 2021. The face of hypervirulent *Klebsiella pneumoniae* isolated from clinical samples of two Iranian teaching hospitals. Ann Clin Microbiol Antimicrob 20:58. doi:10.1186/s12941-021-00467-2. 34465335 PMC8406009

[19] Lee C-R, Lee JH, Park KS, Jeon JH, Kim YB, Cha C-J, Jeong BC, Lee SH. 2017. Antimicrobial resistance of hypervirulent *Klebsiella pneumoniae*: epidemiology, hypervirulence-associated determinants, and resistance mechanisms. Front Cell Infect Microbiol 7:483. doi:10.3389/fcimb.2017.00483. 29209595 PMC5702448

[20] Sanikhani R, Moeinirad M, Shahcheraghi F, Lari A, Fereshteh S, Sepehr A, Salimi A, Badmasti F. 2021. Molecular epidemiology of hypervirulent *Klebsiella pneumoniae*: a systematic review and meta-analysis. Iran J Microbiol 13:257–265. doi:10.18502/ijm.v13i3.6384. 34540163 PMC8416590

[21] Naha S, Sands K, Mukherjee S, Saha B, Dutta S, Basu S. 2021. OXA-181-like carbapenemases in *Klebsiella pneumoniae* ST14, ST15, ST23, ST48, and ST231 from septicemic neonates: coexistence with NDM-5, resistome, transmissibility, and genome diversity. mSphere 6:e01156-20. doi:10.1128/mSphere.01156-20. 33441403 PMC7845606

[22] Fang C-T, Chuang Y-P, Shun C-T, Chang S-C, Wang J-T. 2004. A novel virulence gene in *Klebsiella pneumoniae* strains causing primary liver abscess and septic metastatic complications. J Exp Med 199:697–705. doi:10.1084/jem.20030857. 14993253 PMC2213305

[23] Babraham Institute. 2021. FastQC. https://www.bioinformatics.babraham.ac.uk/projects/fastqc/. Accessed 27 October 2021.

[24] Ewels P, Magnusson M, Lundin S, Käller M. 2016. MultiQC: summarize analysis results for multiple tools and samples in a single report. Bioinformatics 32:3047–3048. doi:10.1093/bioinformatics/btw354. 27312411 PMC5039924

[25] Bolger AM, Lohse M, Usadel B. 2014. Trimmomatic: a flexible trimmer for Illumina sequence data. Bioinformatics 30:2114–2120. doi:10.1093/bioinformatics/btu170. 24695404 PMC4103590

[26] Wood DE, Lu J, Langmead B. 2019. Improved metagenomic analysis with Kraken 2. Genome Biol 20:257. doi:10.1186/s13059-019-1891-0. 31779668 PMC6883579

[27] Bankevich A, Nurk S, Antipov D, Gurevich AA, Dvorkin M, Kulikov AS, Lesin VM, Nikolenko SI, Pham S, Prjibelski AD, Pyshkin AV, Sirotkin AV, Vyahhi N, Tesler G, Alekseyev MA, Pevzner PA. 2012. SPAdes: a new genome assembly algorithm and its applications to single-cell sequencing. J Comput Biol 19:455–477. doi:10.1089/cmb.2012.0021. 22506599 PMC3342519

[28] Boetzer M, Henkel CV, Jansen HJ, Butler D, Pirovano W. 2011. Scaffolding pre-assembled contigs using SSPACE. Bioinformatics 27:578–579. doi:10.1093/bioinformatics/btq683. 21149342

[29] Walker BJ, Abeel T, Shea T, Priest M, Abouelliel A, Sakthikumar S, Cuomo CA, Zeng Q, Wortman J, Young SK, Earl AM. 2014. Pilon: an integrated tool for comprehensive microbial variant detection and genome assembly improvement. PLoS One 9:e112963. doi:10.1371/journal.pone.0112963. 25409509 PMC4237348

[30] Seppey M, Manni M, Zdobnov EM. 2019. BUSCO: assessing genome assembly and annotation completeness. Methods Mol Biol 1962:227–245. doi:10.1007/978-1-4939-9173-0_14. 31020564

[31] Gurevich A, Saveliev V, Vyahhi N, Tesler G. 2013. QUAST: quality assessment tool for genome assemblies. Bioinformatics 29:1072–1075. doi:10.1093/bioinformatics/btt086. 23422339 PMC3624806

[32] Assefa S, Keane TM, Otto TD, Newbold C, Berriman M. 2009. ABACAS: algorithm-based automatic contiguation of assembled sequences. Bioinformatics 25:1968–1969. doi:10.1093/bioinformatics/btp347. 19497936 PMC2712343

[33] Seemann T. 2014. Prokka: rapid prokaryotic genome annotation. Bioinformatics 30:2068–2069. doi:10.1093/bioinformatics/btu153. 24642063

[34] Alcock BP, Raphenya AR, Lau TTY, Tsang KK, Bouchard M, Edalatmand A, Huynh W, Nguyen A-LV, Cheng AA, Liu S, Min SY, Miroshnichenko A, Tran H-K, Werfalli RE, Nasir JA, Oloni M, Speicher DJ, Florescu A, Singh B, Faltyn M, Hernandez-Koutoucheva A, Sharma AN, Bordeleau E, Pawlowski AC, Zubyk HL, Dooley D, Griffiths E, Maguire F, Winsor GL, Beiko RG, Brinkman FSL, Hsiao WWL, Domselaar GV, McArthur AG. 2020. CARD 2020: antibiotic resistome surveillance with the comprehensive antibiotic resistance database. Nucleic Acids Res 48:D517–D525. 31665441 10.1093/nar/gkz935 PMC7145624

[35] Bortolaia V, Kaas RS, Ruppe E, Roberts MC, Schwarz S, Cattoir V, Philippon A, Allesoe RL, Rebelo AR, Florensa AF, Fagelhauer L, Chakraborty T, Neumann B, Werner G, Bender JK, Stingl K, Nguyen M, Coppens J, Xavier BB, Malhotra-Kumar S, Westh H, Pinholt M, Anjum MF, Duggett NA, Kempf I, Nykäsenoja S, Olkkola S, Wieczorek K, Amaro A, Clemente L, Mossong J, Losch S, Ragimbeau C, Lund O, Aarestrup FM. 2020. ResFinder 4.0 for predictions of phenotypes from genotypes. J Antimicrob Chemother 75:3491–3500. doi:10.1093/jac/dkaa345. 32780112 PMC7662176

[36] Maiden MCJ, Jansen van Rensburg MJ, Bray JE, Earle SG, Ford SA, Jolley KA, McCarthy ND. 2013. MLST revisited: the gene-by-gene approach to bacterial genomics. Nat Rev Microbiol 11:728–736. doi:10.1038/nrmicro3093. 23979428 PMC3980634

[37] Lam MMC, Wick RR, Watts SC, Cerdeira LT, Wyres KL, Holt KE. 2021. A genomic surveillance framework and genotyping tool for *Klebsiella pneumoniae* and its related species complex. Nat Commun 12:4188. doi:10.1038/s41467-021-24448-3. 34234121 PMC8263825

[38] Treangen TJ, Ondov BD, Koren S, Phillippy AM. 2014. The Harvest suite for rapid core-genome alignment and visualization of thousands of intraspecific microbial genomes. Genome Biol 15:524. doi:10.1186/s13059-014-0524-x. 25410596 PMC4262987

[39] Kaas RS, Leekitcharoenphon P, Aarestrup FM, Lund O. 2014. Solving the problem of comparing whole bacterial genomes across different sequencing platforms. PLoS One 9:e104984. doi:10.1371/journal.pone.0104984. 25110940 PMC4128722

[40] Antipov D, Hartwick N, Shen M, Raiko M, Lapidus A, Pevzner PA. 2016. plasmidSPAdes: assembling plasmids from whole genome sequencing data. Bioinformatics 32:3380–3387. doi:10.1093/bioinformatics/btw493. 27466620

[41] Alikhan N-F, Petty NK, Ben Zakour NL, Beatson SA. 2011. BLAST Ring Image Generator (BRIG): simple prokaryote genome comparisons. BMC Genomics 12:402. doi:10.1186/1471-2164-12-402. 21824423 PMC3163573

[42] Letunic I, Bork P. 2019. Interactive Tree of Life (iTOL) v4: recent updates and new developments. Nucleic Acids Res 47:W256–W259. doi:10.1093/nar/gkz239. 30931475 PMC6602468

[43] Li H, Durbin R. 2009. Fast and accurate short read alignment with Burrows-Wheeler transform. Bioinformatics 25:1754–1760. doi:10.1093/bioinformatics/btp324. 19451168 PMC2705234

[44] Ramírez F, Dündar F, Diehl S, Grüning BA, Manke T. 2014. deepTools: a flexible platform for exploring deep-sequencing data. Nucleic Acids Res 42:W187–W191. doi:10.1093/nar/gku365. 24799436 PMC4086134

[45] Van der Auwera GA, Carneiro MO, Hartl C, Poplin R, Del Angel G, Levy-Moonshine A, Jordan T, Shakir K, Roazen D, Thibault J, Banks E, Garimella KV, Altshuler D, Gabriel S, DePristo MA. 2013. From FastQ data to high confidence variant calls: the Genome Analysis Toolkit best practices pipeline. Curr Protoc Bioinformatics 43:11.10.1–11.10.33. doi:10.1002/0471250953.bi1110s43. PMC4243306 25431634

[46] Cingolani P, Platts A, Wang LL, Coon M, Nguyen T, Wang L, Land SJ, Lu X, Ruden DM. 2012. A program for annotating and predicting the effects of single nucleotide polymorphisms, SnpEff: SNPs in the genome of Drosophila melanogaster strain w1118; iso-2; iso-3. Fly (Austin) 6:80–92. doi:10.4161/fly.19695. 22728672 PMC3679285

[47] Gu D, Dong N, Zheng Z, Lin D, Huang M, Wang L, Chan EW-C, Shu L, Yu J, Zhang R, Chen S. 2018. A fatal outbreak of ST11 carbapenem-resistant hypervirulent *Klebsiella pneumoniae* in a Chinese hospital: a molecular epidemiological study. The Lancet Infectious Diseases 18:37–46. 28864030 10.1016/S1473-3099(17)30489-9

