# Supplementary material for: Comparative Analysis of Clinical and Genomic Characteristics of Hypervirulent Klebsiella pneumoniae from Hospital and Community Settings: Experience from a Tertiary Healthcare Center in India
Source: Microbiol Spectr. 2022 Aug 31;10(5):e00376-22. doi: 10.1128/spectrum.00376-22 (PMC9602566; doi:10.1128/spectrum.00376-22)
Supplement: Supplemental file 1 — Tables S1 to S3. Download spectrum.00376-22-s0001.pdf, PDF file, 0.1 MB [file spectrum.00376-22-s0001.pdf]

Table S1: Genome quality statistics for all the assembled genomes

Isolate no.	No. of contigs	Largest contig	Size(bp)	GC (%)	N50	L50	Genome fraction (%)
S44	40	517772	5482928	57.27	308712	7	92.224
S54	46	1082409	5691332	57.15	435362	4	98.04
S55	79	1010433	5752447	56.75	345690	6	92.898calc
S56	86	992865	5708668	57.05	226397	5	90.508
S57	101	605472	5718919	56.86	233432	9	90.207
S58	104	584373	5839331	56.58	306551	8	91.983
S59	70	698263	5769731	56.77	350190	6	93.125
S60	72	851986	5714482	56.83	286886	6	93.1
S61	34	512126	5352578	57.8	363661	7	33.733
S62	2947	86358	7457857	56.15	4818	346	92.984
S63	2187	71415	7190142	56.28	11256	176	93.917
S65	83	698263	5745130	56.78	287340	7	93.105
S66	335	357715	6494540	56.07	95700	19	93.42
S67	115	504304	6062433	56.53	200969	11	93.098

Table S2: Sequence types of isolates based on MLST scheme

Isolate	Chr_ST	gapA	infB	mdh	pgi	phoE	rpoB	tonB
S44	ST86	9	4	2	1	1	1	27
S54	ST23	2	1	1	1	9	4	12
S55	ST2096	1	6	1	1	1	46	1
S56	ST147	3	4	6	1	7	4	38
S57	ST43	2	6	1	5	11	1	15
S58	ST231	2	6	1	3	26	1	77
S59	ST2096	1	6	1	1	1	46	1
S60	ST2096	1	6	1	1	1	46	1
S61	ST3857	18	23	56	260	25	92	548
S62	ST147-1LV	3	4	6	1	-	4	38
S63	ST336-1LV	2	1	1	1	72	4	-
S65	ST2096	1	6	1	1	1	46	1
S66	ST15	1	1	1	1	1	1	1
S67	ST15-1LV	1	1	1	1	1*	1	1

Data Availability:

The genome sequenced files are submitted to NCBI SRA under the accession number SRR17640814, SRR17640813, SRR17640812, SRR17640811, SRR17640810, SRR17640809, SRR17640808, SRR17640807, SRR17640806, SRR17640805, SRR17640804, SRR17640803, SRR17640802, SRR17640801

Table S3: List of highly mutated genes with their respective functions (as found in UniProt and NCBI)

Gene Names	Function
KP1_0403	Putative secretion ATPase
KP1_4102	hypothetical protein
fimD	Putative fimbrial usher protein
mobB	Mobilization protein, GTP binding
wzi	Surface assembly of capsule
wzc	ATP Binding and protein tyrosine kinase activity
KP1_0410	Fimbrial biogenesis outer membrane usher protein
ardC	Single-stranded DNA binding
wza	Polysaccharide transmembrane transporter activity
KP1_4970	AsmA2 domain-containing protein YhdP
RmpA	DNA binding
WcaJ	Transferase activity
entA	2,3-dihydro-2,3-dihydroxybenzoate dehydrogenase activity
entD	holo-[acyl-carrier-protein] synthase activity, magnesium ion binding
entC	isochorismate synthase activity
